# Bridging Quantum Mechanics and Biology at the Million-Atom Scale

**DOI:** 10.21203/rs.3.rs-7327472/v1

**Published:** 2025-09-16

**Authors:** Luc Wieners, Martin E. Garcia

**Affiliations:** 1 Institute of Physics, University of Kassel, Heinrich-Plett-Straße 40, 34132 Kassel, Germany

## Abstract

The behaviour of proteins and other biomolecules is mainly governed by the quantum-mechanical character of their electrons. Accurately capturing the resulting interactions is essential for predicting molecular properties, obtaining spectroscopic data, and advancing drug design. However, the extreme computational cost of quantum calculations has historically limited their application to small systems of just a few hundred atoms. Here, we present a quantum-mechanical method that enables electronic structure calculations on biological systems at unprecedented scales, up to millions of atoms, while drastically reducing computational costs. We apply this approach to entire proteins and large biomolecular assemblies, including a complete bacteriophage^1^ in a solution containing over 150 million electrons. Additionally, we show that atomic energies computed for AlphaFold-predicted protein structures strongly correlate with AlphaFold’s confidence scores^2^, providing a new quantum-based validation metric. The method’s efficiency also allows the accurate prediction of spectroscopic properties for biomolecules previously out of reach for first-principles techniques. We present computed spectra for DNA^3,4^ and the anticancer drug Actinomycin^5,6^, involving hundreds to thousands of atoms, in close agreement with experimental measurements. This advance bridges quantum mechanics and biology at a previously inaccessible scale, enabling large-scale, first-principles simulations with broad applications in quantum biology, structural biology, medicine, and materials science.

Biomolecules, including proteins, nucleic acids and large assemblies, underpin nearly all processes of life. The rich range of their structures and functions arises from their ability to undergo conformational transitions and biochemical reactions, the latter often involving the formation or breaking of covalent bonds and the redistribution of electrons. While classical molecular mechanics methods^7,8,9^ can successfully describe large-scale conformational changes, a fully accurate treatment of processes that require bond rearrangements, such as enzyme catalysis, photoexcitation, or protein production, must account for the quantum nature of electrons. To tackle these problems, hybrid quantum mechanics/molecular mechanics (QM/MM) methods^10,11^ have become a popular compromise, applying a quantum description to a small reactive site while retaining classical models elsewhere. However, this approach cannot fully capture the rich electronic effects that emerge across large biomolecular assemblies. A fully quantum-mechanical treatment at this scale has remained elusive, largely due to prohibitive computational cost and unfavourable scaling of standard algorithms.

Here we present a scalable framework that makes it possible to perform first-principles electronic structure calculations^12,13^ on whole proteins and large biomolecular^14,15,16,17,18,19,20,21^ complexes. Our approach is based on the all-electron Hartree–Fock method and comprises two key innovations. First, we identify and discard those Coulomb integrals that have a negligible influence on the final results, thereby reducing computational effort without compromising accuracy. Second, we employ a divide-and-conquer strategy to partition large systems into manageable segments, allowing us to treat their electronic structure quantum-mechanically at unprecedented scale (see Figure 8).

This framework opens up a path toward fully quantum-mechanical simulations of complex biomolecular systems, the possibility of shedding light on their mechanisms, properties, and functions, and paves the way for future applications in structural biology, biochemistry, drug design and interaction of external electric, magnetic and electromagnetic fields with proteins and DNA.

## Quantum divide & conquer in biomolecules

To enable all-electron quantum-mechanical calculations on large biomolecular systems, we implemented a divide-and-conquer strategy^15,19,22^ that avoids the unfavourable scaling of conventional methods. Instead of solving the Hartree–Fock equations for the whole molecule at once, which typically scale cubically with system size, we split the system into overlapping clusters of manageable size. Each of these clusters is treated separately in a Hartree–Fock framework, yielding an order-N scaling of computational effort^12,16,17,22,23,24,25,26,27^ and allowing the procedure to be easily parallelized across multiple processors. Every cluster comprises a central “core” region containing the atoms of primary interest, while a surrounding “buffer” region captures the effects of its environment. Only the electron density of the core is kept for assembling the total density, while the buffer guarantees that boundary effects and long-range interactions are adequately represented. The clusters are then smoothly merged by interpolating their density matrices, yielding a coherent and accurate electron density for the entire system (see Methods).

**Figure 1:**
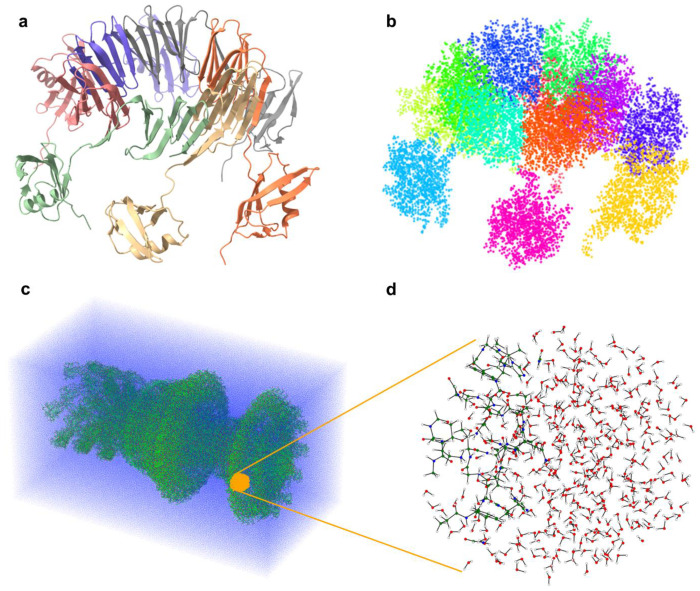
Divide-and-conquer clustering for linear-scaling quantum calculations. **a**, The protein complex (PDB 2M3X)^28^ is shown as an example system. **b,** The molecule is spatially partitioned into 12 clusters using the k-means algorithm. **c**, For larger systems in a solution, such as a flagellar motor in water, the individual clusters are obtained by subdividing the system using a three-dimensional grid. **d**, Corresponding grid-based clusters are shown. Visualisation of the protein structure in **a** was done with ChimeraX^29^.

To generate the subsystems used in the divide-and-conquer scheme, we apply different strategies, depending on the structural characteristics of the biomolecule. For heterogeneous systems with large empty regions we employ the k-means clustering algorithm to group nearby atoms into spatially localized clusters that serve as the core regions for quantum calculations (see Figure 1b). On the other hand, for more homogeneous systems (as for example protein complexes in a solution (Figure 1c)), we use a three-dimensional grid to define the partitioning.

Once the core regions are identified, we expand each cluster by adding surroundings of atoms within a fixed cut-off distance (typically of 8–10 Å), to form the buffer region which captures the long-range effects. However, adding atoms just based on their distance often leads to an artificial cutting of covalent bonds, introducing unphysical boundary conditions. To overcome this problem, we apply chemically informed rules: if a double bond is intersected, both bonded atoms are included in the cluster. For a single bond, the cut atom is replaced by a hydrogen atom positioned at the typical length of the bond X-H for a corresponding atom of species X. Bond character is determined from atomic distances, ensuring that all cluster boundaries preserve chemically realistic connectivity.

Achieving linear scaling is essential for large-scale quantum-mechanical calculations. But to make such calculations practical, it is also important to reduce the prefactor of the now linear complexity curve as much as possible. This requires not only efficient partitioning but also highly optimized algorithms for performing the Hartree–Fock all-electron calculations within each subsystem. We therefore developed new screening algorithms^30^ which estimate the relevance of the interaction of two wave functions (which together form an electronic density) based on their overlap. In addition to this, we use a cut-off for the Coulomb interaction of two densities (for example 10 A). In combination these methods drastically reduce the number of evaluated two-electron repulsion integrals (ERIs) which usually are the computational bottleneck of Hartree–Fock calculations, since they scale with the fourth power of the system size.

To illustrate the efficiency gains of our method, we applied it to the retinal protein Rhodopsin (PDB 1H68)^31^, which consists of 3,439 atoms. In the STO-3G basis set, the theoretical number of ERIs is approximately 1.06·10^16^, of which 1.33 10^15^ have distinct values. By applying a filtering scheme based on relevant densities (see Methods), we reduced the number of significant ERIs to 1.57·10^11^. A second filtering step, specific to the Coulomb interaction, further reduced this value to just 1.33·10^9^ ERIs, a decrease of six orders of magnitude with respect to the full set. For the efficient computation of the remaining ERIs, we used a Gaussian lobe function expansion^32^, which enables highly parallelizable algorithms and contributes to the overall scalability of the method.

In addition to the computation time, memory requirements are a critical factor in Hartree–Fock calculations. The need to store the ERIs usually presents the main memory bottleneck, as even a single system may easily involve more than several billion ERIs, exceeding the storage capacity of typical high-performance computing nodes. Our filtering strategy effectively overcomes this limitation by discarding integrals that negligibly contribute to the final result, dramatically reducing memory usage while preserving accuracy.

When applying the divide-and-conquer strategy to systems with more than 100,000 atoms^14,15,22,27^, the number of subsystems can itself become a limiting factor, particularly in terms of memory requirements on high-performance computing facilities. Because subsystems interact, their data must remain accessible to one another, typically requiring all clusters to be evaluated in parallel, which can overwhelm available resources. We overcome this problem by adding controlled overlaps between neighbouring subsystems, allowing them to be processed sequentially rather than simultaneously. This innovation removes a major scaling bottleneck (see Methods) and enables quantum-mechanical calculations on systems with over 10 million atoms, a size previously considered intractable.

## Electronic structure of a bacteriophage

Using our modified Hartree–Fock formalism, we performed quantum-mechanical calculations on entire biophysical systems comprising millions of atoms, including, to our knowledge, the largest system ever treated at this level of theory. As benchmarks, we selected three structurally distinct complexes in explicit water: a bacterial flagellar motor (PDB 8WL2)^33^, a vault ribonucleoprotein particle (PDB 7PKR, see Figure 6)^34^ and a complete Staphylococcus aureus bacteriophage (P68, PDB 6Q3G)^1^. These systems consist of approximately 4.37, 13.5 and 45.1 million atoms, respectively. This corresponds to 14.9, 45.4 and 151.3 million electrons, respectively, including those of the water molecules.

**Figure 2:**
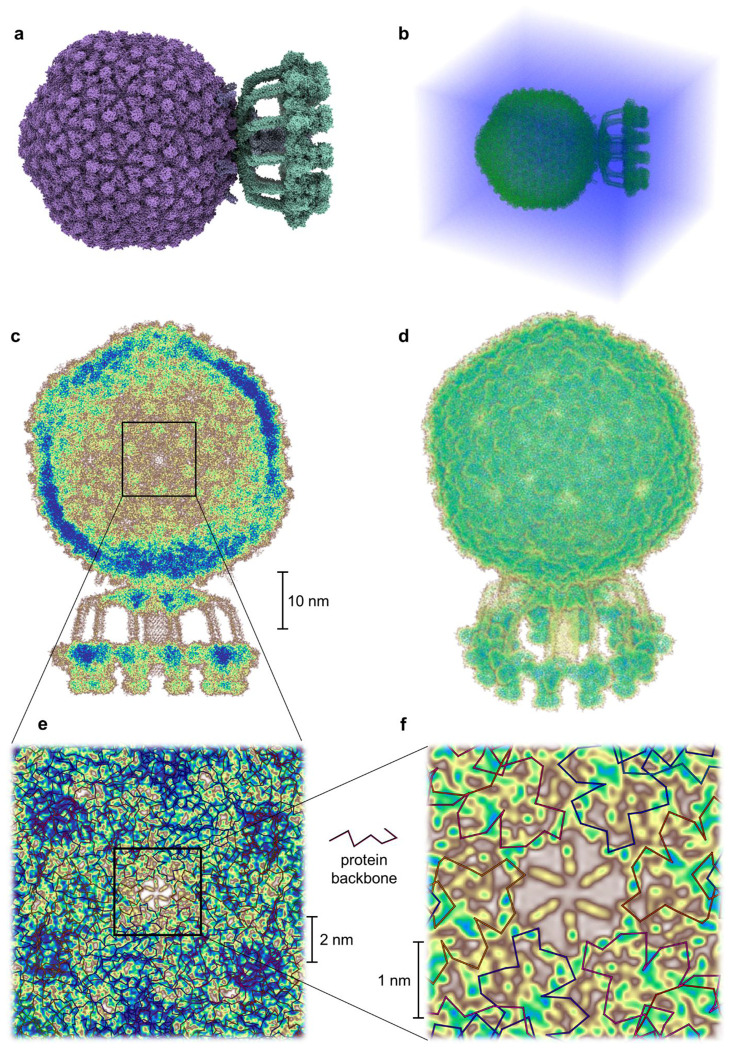
Visualisation of the modified all-electron Hartree–Fock—exemplified on the bacteriophage P68^1^ in a solution of water. **a,b**, Atomic-scale resolved structure of the bacteriophage itself and in solution. **c,d,** Calculated electronic density as a projection along the x-axis and as a three-dimensional picture (see also Figure 10). **e,f**, Close-up of the projected electronic density of **c** after successive enlargements. Brown corresponds to low and blue to high electronic density values. For a better visualisation the electronic density of the solvent is not displayed and only valence basis function contributions are shown. Visualisation of the protein structure in **a** was done with ChimeraX^29^.

The bacteriophage P68 comprises 668 protein subunits, together containing 133,777 amino acids (2,148,795 atoms). The structure was solvated in a water box extending 10 nm beyond the molecular surface in all directions, yielding a total system size of 67.2 × 78.8 × 86.8 nm^3^. To enable the calculation, we divided the system into 241,920 overlapping subsystems, each with a central region of (12.5 Å)^3^ and 8 Å overlap. Hartree–Fock calculations were carried out independently for each subsystem, and the global electronic density was reconstructed by interpolating the nonzero elements of the subsystem density matrices. Orbital energies were also retained for subsequent analysis. The full calculation was performed on 150 compute nodes (Intel Xeon Platinum 9242, 96 cores/node) and completed in 12 hours, amounting to 173,000 CPU core hours and approximately 3.2·10^19^ double-precision floating-point operations (32 exaFLOPs). A uniform grid with 1.5 Bohr spacing was used to evaluate the total electron density, shown in Figure 2.

To make this scale of computation possible, we used the STO-3G basis set, which offers a favourable trade-off between qualitative accuracy and computational efficiency. Compared to larger split-valence and polarisation basis sets, our approach reduces the number of electron repulsion integrals by orders of magnitude, significantly lowering memory requirements and diagonalization time. Relative to state-of-the-art high-accuracy Hartree–Fock implementations^14^, we achieve a computational speedup of more than three orders of magnitude in FLOPs per atom.

## Quantum optical spectra of biomolecules

A defining strength of quantum-mechanical approaches is their ability to predict optical properties that are inaccessible to classical models. Absorption spectra, for example, arise from electronic excitations and therefore require a fully quantum description of the electronic structure. Using our Hartree–Fock framework in its real-time time-dependent Hartree–Fock (RT-TDHF) implementation^35^, an approach that offers more favourable scaling for large systems than standard linear-response methods, we computed UV/Vis absorption spectra^13^ of large biomolecules^36^. Thanks to efficient matrix operations and integral filtering in the STO-3G basis, we are able to calculate the optical response of systems comprising several hundred to a few thousand atoms, a regime typically out of reach for direct quantum spectral calculations.

**Figure 3:**
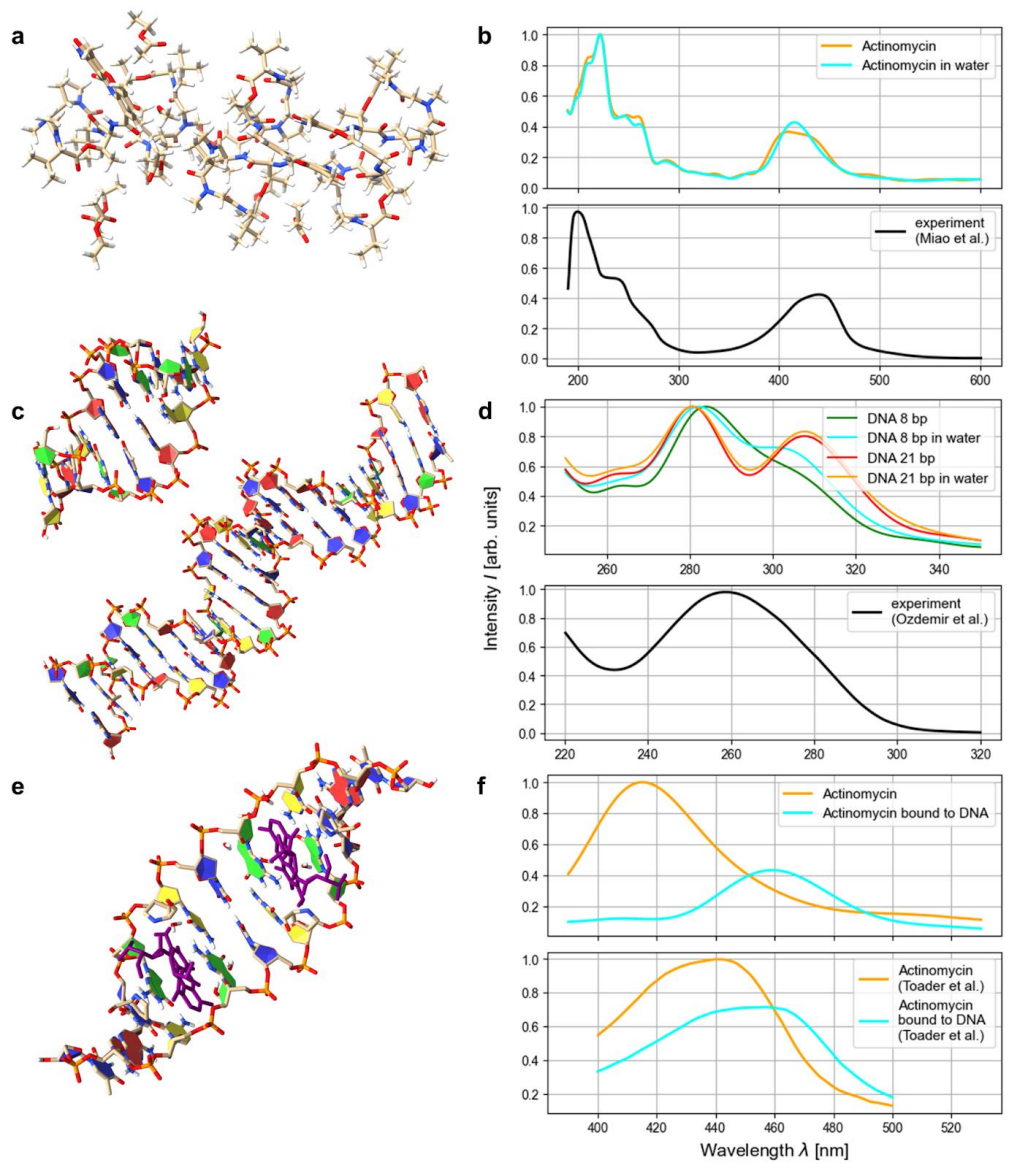
Computed spectra of biomolecules in comparison with experiments. Absorption spectra were calculated for Actinomycin^5^ (**a**), DNA with 8 and 21 base pairs^3,4^ (**c**) and Actinomycin bound to DNA^6^ (**e**) and compared to experimental data from Miao et al.^37^ (**b**), Ozdemir et al.^38^ (**d**) and Toader et al.^39^ (**f**). Visualisation of the biomolecular structures in **a**, **c** and **e** was done with ChimeraX^29^.

We applied this method to obtain the absorption spectra of DNA^3,4^ and the anticancer drug Actinomycin D^5^ as well as a DNA-Actinomycin D complex^6^ and compared to experimentally obtained spectral data^37,38,39^ (see Figure 3). These systems are—to our knowledge—the largest systems for which theoretical UV/Vis spectral data has been obtained from first principles.

## Quantum energies and confidence scores

AlphaFold^2^ has revolutionized protein structure prediction, offering remarkably accurate models accompanied by residue-level confidence scores known as pLDDT (predicted local distance difference test) value^40^. These scores range from 0 (high uncertainty) to 100 (high confidence), offering an estimate of how closely a predicted structure matches the true, native conformation.

Using our linear-scaling Hartree–Fock approach, we calculate atomic energies of entire protein structures efficiently and with quantum-mechanical accuracy. Because proteins tend to fold into structures that minimize their free energy^41^, local atomic energies provide a direct, physics-based measure of structural stability. Regions with low atomic energies are expected to be close to their native, folded state, whereas high-energy regions are a sign of instability or disorder. This allows us to obtain a measure for the assessment of protein structures based on first-principles methods (see Figure 7).

**Figure 4:**
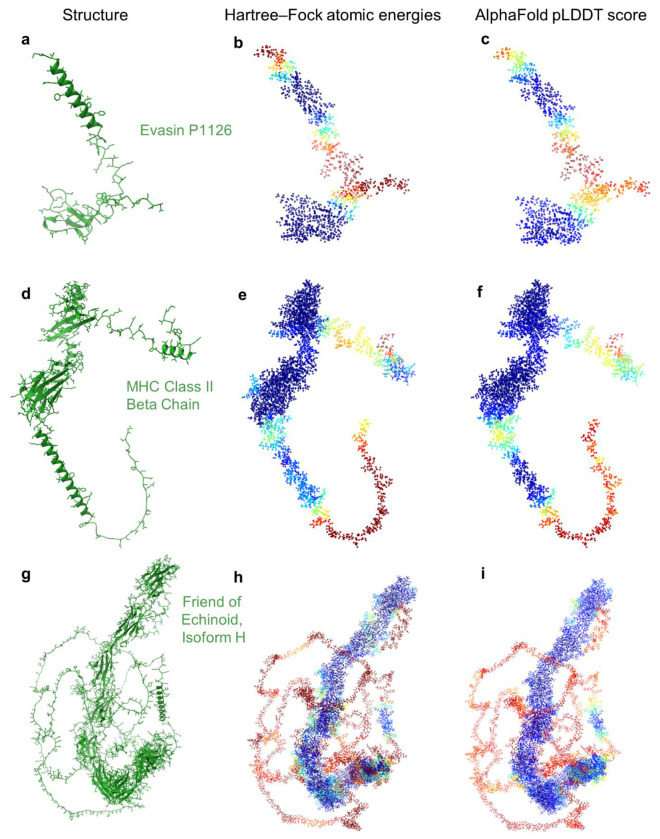
Comparison of quantum-mechanical atomic energies with AlphaFold confidence scores. Atomic energies from Hartree–Fock calculations are compared with AlphaFold’s pLDDT score for three predicted protein structures: Evasin P1126 (PDB AF_AFA0A023FF81F1) (**a**), MHC Class II Beta Chain (AF_AFA0A023IKK2F1) (**b**) and Friend of Echinoid, Isoform H (AF_AFA0A023GPK8F1) (**c**). For each protein, the left column shows ribbon diagrams and non-hydrogen atoms. The middle column (**b,e,h**) displays atomic energies computed in a solvated environment (10 Å water shell), smoothened using a Savitzky–Golay filter, scaled by element number, and linearly rescaled to match the pLDDT range. The right column (**c,f,i**) shows the corresponding AlphaFold pLDDT scores mapped onto atoms. Energies of outlier atoms were clipped to match the [0,100] range of pLDDT values. Protein structures in **a, d** and **g** were visualized using ChimeraX^19^. Data from rcsb.org^42^.

To evaluate this principle, we studied three AlphaFold-predicted proteins: Evasin P1126 (PDB AF_AFA0A023FF81F1), MHC Class II Beta Chain (PDB AF_AFA0A023IKK2F1), and Friend of Echinoid Isoform H (PDB AF_AFA0A023GPK8F1), obtained from the RCSB PDB^42^ and containing 90, 287, and 1,447 amino acids, respectively. Each structure was solvated with a 10 Å shell of water to create a more realistic environment and to avoid artificial edge effects. The resulting system sizes ranged from ~9,000 to over 115,000 atoms, with corresponding runtimes from under an hour to 14 hours on a single compute node. Atomic energies were calculated and normalized by element type (using only valence basis functions) and further smoothened using a suitable filter to remove short-range noise (see Methods). These energies were rescaled and clipped to match the [0,100] range of pLDDT scores, enabling a direct comparison. Strikingly, we found a strong correlation between quantum-mechanical atomic energies and AlphaFold’s pLDDT scores across all three proteins (Figure 4). Regions with low energies aligned closely with AlphaFold’s high-confidence predictions, while higher energies corresponded to lower pLDDT values. A similar trend was observed when using simplified energy models based on ionic contributions alone, although with reduced accuracy. In comparison to classical heuristics such as counting neighbouring atoms to evaluate protein structures via atom densities, the quantum-mechanical approach exhibited a markedly stronger correlation with pLDDT scores, stressing the value of using first-principles methods in assessing protein folding predictions (see Figure 9).

Our results suggest that AlphaFold’s predictions not only approximate correct protein structures, but also implicitly capture aspects of the quantum-mechanical energy landscape—despite not being trained on quantum data. This opens the door to using first-principles calculations to validate and interpret machine-learning-based structure predictions and provides an independent, physics-grounded confidence metric.

## Discussion

The calculation of atomic energies via the presented modified Hartree–Fock scheme offers a physically grounded metric for assessing protein structure predictions, independent of machine learning biases. Given its high computational efficiency, this approach provides a complementary method to evaluate predicted models—especially in regions with limited training data or in intrinsically disordered proteins^43^. The ability to localize energetically unfavourable regions could support the identification of misfolded domains or regions of structural uncertainty.

Beyond structure assessment, the fast-running RT-TDHF algorithm enables the calculation of optical absorption spectra for biomolecular systems previously inaccessible to ab initio methods. This includes drug molecules and their interactions with DNA or proteins, as demonstrated for Actinomycin D. For smaller molecules, the low computational requirements of the modified Hartree–Fock algorithm even permit UV/Vis spectra to be calculated on personal computers, potentially eliminating the need of high-performance computing in many cases and broadening access to theoretical spectroscopy.

**Figure 5:**
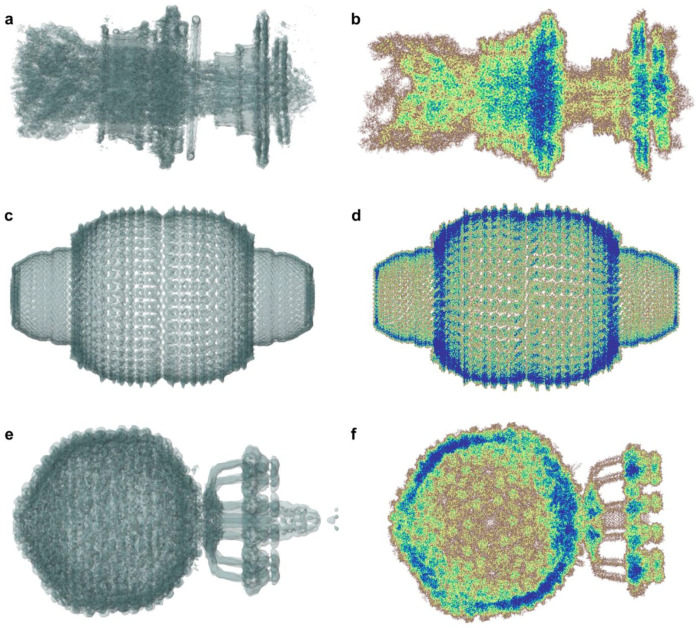
Cryo-EM and computed densities. Comparison of electronic densities determined via cryogenic electron microscopy (**a**,**c**,**e**) with quantum-mechanically computed electronic densities (**b**,**d**,**f**) for a flagellar motor (PDB 8WL2)^33^, a vault protein complex (PDB 7PKR)^34^ and a bacteriophage (PDB 6Q3G)^1^. **a**,**c**,**e** were visualised using the Mol* Viewer^85^ from rcsb.org^42^.

In addition, the direct access to the electronic density enables further applications^44^. Dipole moments, for instance, can be determined with higher accuracy than via classical approximations ^45^. More importantly, the computed electronic densities allow direct comparison with x-ray diffraction experiments, making it possible to theoretically reconstruct electron density maps at quantum-mechanical precision (see Figure 5). This is particularly relevant for emerging high-resolution crystallographic methods (<1 Å), where conventional density-fitting techniques often fall short in capturing deformation effects^46^.

Finally, the ability to compute electronic structures for systems comprising millions of atoms facilitates the generation of large-scale quantum-accurate datasets, an asset for machine-learning applications^47^. By moving beyond small-model systems thanks to the increased computational speed, such datasets may help reduce biases in training and enable broader generalisation in ML-based property predictions.

Overall, the combination of Hartree–Fock theory with a divide-and-conquer strategy and additional algorithms opens a new regime for large-scale quantum simulations with drastically reduced runtimes and computational resources. In addition to the applications explored here, future directions include first-principles molecular dynamics without QM/MM partitioning, calculations under external electric or magnetic fields, and description of excited-state or bond-breaking processes. These developments open possibilities in quantum biology, such as investigating the microscopic mechanisms of photosynthesis, understanding the effects of magnetic fields on protein and DNA dynamics, modelling radiation-induced damage at the atomic level, or simulating light-induced conformational changes in chromophoric proteins. Finally, the integration of quantum-accurate datasets with machine-learning techniques may further accelerate the discovery and design of biomolecular functions across biology, medicine, and materials science.

## Methods

### Modifications to the Hartree–Fock algorithm

Unlike density functional theory (DFT), Hartree–Fock is a wavefunction-based method and does not directly operate on the electronic density. Each electronic interaction integral contains products of four wave functions. As the system grows, the amount of involved wave functions increases, and the number of electronic repulsion integrals (ERIs) scales with the fourth power of the system size, making their evaluation one of the main computational bottlenecks of Hartree–Fock calculations. To address this, we introduce systematic approximations that significantly reduce the computational effort. Both wavefunction-wavefunction ^30^ and density-density interactions are truncated using physically motivated thresholds. Since wave functions decay exponentially with distance, only nearby orbitals contribute significantly to overlaps (see subsection “Density cut-offs”). We assess the relevance of a density by calculating the overlap of its two constituent wave functions. For orbital types with negative signs, we use the absolute of the product to capture significant interactions that may otherwise cancel numerically but still produce meaningful contributions, especially to energy gradients, which are important for force calculations. Once densities below the threshold are excluded, the scaling of ERI evaluations drops from quartic to quadratic. To further reduce complexity to linear scaling (order N), we introduce a cut-off for the Coulomb interaction based on inter-density distance (see subsection “Coulomb cut-offs”). Since electrostatic interactions decay with the inverse of the distance between two charges, we use a Coulomb cut-off between 8 and 12 Å, similar to that employed in classical forces fields for biophysical simulations. The distance of two densities is defined as the distance between the centres of the two wavefunction pairs that generate them. Only ERIs with Coulomb distances below the Coulomb cut-off are calculated. For ERIs with distances near the cut-off, we apply a smoothening function to ensure continuity.

An ERI is evaluated if the overlap threshold of both densities is exceeded and their centres lie within the Coulomb cut-off. Additionally, we compute the product of the two density relevance values (see the next subsections), divide it by the distance between their centres, and compare it to a final relevance threshold. This avoids evaluating interactions between weakly overlapping densities and/or those separated by large distances. To further accelerate the process, we adopt an efficient and parallelizable ERI algorithm^48,49,50^ based on the so-called Gaussian lobe functions^32,51^ (see subsection “Basis function fitting”). Unlike conventional approaches^52,53,54,55^, which use angular prefactors (x, y, z) to construct directional orbitals, our method builds positive and negative lobes from distinct Gaussian components. This removes angular dependencies and simplifies the integral expressions, making them uniform across orbital types. As a result, all ERI calculations can be performed by using the same expression, which enables a simple parallelisation for both ERIs and their derivatives (necessary to calculate atomic forces). We also precompute components of the ERIs which only depend on pairs of wave functions. This reduces the full calculation of the electronic repulsion between two densities consisting of in total of four Gaussian functions to only 6 additions, 8 multiplications, 10 memory accesses, 1 square root and the evaluation of the error function (see below). For most interactions with large separations, the error function can be approximated as unity, further reducing computational cost.

The following subsections detail the generation of the lobe-based basis functions, the calculation of the density relevance, the calculation of the Coulomb interaction relevance and the mathematical expressions for the modified ERI algorithm.

### Basis function fitting

In electronic structure calculations, wave functions are commonly modelled as Slater-type orbitals (STOs), which for an s-orbital have the form $\varphi_{i}(r)=A_{i}\exp\left(-\alpha_{i}\left\|r-r_{i}\right\|\right)$. To enable efficient integration, STOs are typically approximated by using Gaussian functions of the form $g_{i}(r)=A_{i}exp\left(-\alpha_{i}{\left\|r-r_{i}\right\|}^{2}\right)$.

Each STO is approximated by a linear combination of $n_{G}$ Gaussians as

(1) $$\varphi_{i}(r)=\sum_{a=1}^{n_{{\text{G}}_{i}}}A_{a}\text{exp}\left(-\alpha_{a}{\left\|r-r_{a}\right\|}^{2}\right),$$

This approximation enables an analytic evaluation of key integrals^52,53,54,55^, relevant for the overlap matrix, the kinetic and nuclei energy as well as the Coulomb and exchange interaction of the electrons in the system. Orbitals with higher angular momenta are typically expressed via Gaussians containing cartesian prefactors (*x*, *y*, *z*, *x*^2^, *xy*, *xz*, …). Although these integrals can also be evaluated analytically, their complexity increases due to the recursive nature of the associated expressions^52,53,54,55^. Furthermore, different angular momentum combinations require distinct equations, reducing parallelisation efficiency.

To avoid this bottleneck, we here adopt Gaussian lobe function expansions^32,51^, which allow the construction of basis sets without cartesian prefactors. We show in this work that this approach, though currently not widely used, is particularly well-suited to modern computing architectures optimized for high parallelisation. It also enables the evaluation of ERIs using a unified matrix formalism, which is potentially compatible with GPU acceleration^56^. The algorithms for ERI evaluation are described in the section “Electron repulsion integral algorithm”.

For fitting, we start with a Slater-type orbital of the STO-6G type taken from the Basis Set Exchange^57^. In the case of p-orbitals, we approximate them with 6 Gaussian functions, three paced symmetrically slightly to the left and three to the right of the orbital centre. This corresponds in accuracy to a conventional STO-3G orbital. The fitting is performed on a three-dimensional real space grid. Fit parameters include 3 contraction exponents and 3 coefficients, with the latter multiplied by −1 on one side to model the nodal character of p-orbitals. For the optimisation of the parameters during the fitting process, the trust region algorithm is used. This is done via the function curve_fit from the mathematic Python library scipy^58^.

### Density cut-offs

A density $\rho_{ij}(r)$ is defined as the product of two wave functions:

(2) $$\rho_{ij}(r)=\varphi_{i}(r)\varphi_{j}(r)=\sum_{a=1}^{n_{{\text{G}}_{i}}}\sum_{b=1}^{n_{{\text{G}}_{j}}}A_{a}A_{b}\text{exp}\left(-\alpha_{a}{\left\|r-r_{a}\right\|}^{2}-\alpha_{b}{\left\|r-r_{b}\right\|}^{2}\right).$$

To determine whether a given density is relevant for further calculations, we compute a scalar value $r\left(\rho_{ij}\right)$, given by the integral over the absolute value of all pairwise products of Gaussian functions:

(3) $$r\left(\rho_{ij}\right)=\int dr\sum_{a=1}^{n_{{\text{G}}_{i}}}\sum_{b=1}^{n_{{\text{G}}_{j}}}\left|A_{a}\right|\left|A_{b}\right|\text{exp}\left(-\alpha_{a}{\left\|r-r_{a}\right\|}^{2}-\alpha_{b}{\left\|r-r_{b}\right\|}^{2}\right).$$

This equation is valid for s-type orbitals, where the integrand remains positive. Orbitals with nonzero angular momentum (e.g. p-orbitals) include prefactors *x*, *y*, *z* which would require using the absolute values |x|,|y|,|z| to ensure that the integrand is always positive. However, using these absolute values makes an analytic evaluation of the overlap integrals impossible. To resolve this, we developed an approach to represent each lobe of orbitals with a separate set of Gaussians. The previously described lobe-based basis functions (which achieve a nodal plane by summing Gaussians of different signs) are not suitable here, as their absolute-value integrals do not correspond to the absolute value of the full orbital. Instead, we independently fit each lobe of a p-type orbital using Gaussian functions, and then reconstruct the full orbital by combining lobes with different signs and different centres. The representation of a p-type orbital used for the density relevance calculations is given by:

(4) $$\varphi_{i}(r)=\sum_{l=1}^{n_{{\text{L}}_{i}}}\sum_{a=1}^{n_{{\text{G}}_{i}}}s_{l}A_{a}\cdot \text{exp}\left(-\alpha_{x_{i,a}}{\left(x-x_{0_{i,l,a}}\right)}^{2}-\alpha_{y_{i,a}}{\left(y-y_{0_{i,l,a}}\right)}^{2}-\alpha_{z_{i,a}}{\left(z-z_{0_{i,l,a}}\right)}^{2}\right)$$

where $s_{l}$ is the sign of lobe l and each cartesian direction has an individual contraction factor (e.g. $\alpha_{x_{i,a}}$) and offset (e.g. $x_{0_{i,l,a}}$) for higher flexibility upon fitting. The fitting process is identical to that described in the section “Basis function fitting”.

### Coulomb cut-offs

In the Hartree–Fock formalism, the Coulomb interaction is modelled as the repulsion between two electronic densities. Following the method described above, Coulomb interactions are neglected if the separation between densities exceeds a defined cut-off. However, since densities are not point-like, a suitable distance metric must be established to estimate their effective separation. For this purpose, each density is assigned an origin, calculated as the arithmetic mean of the atomic positions associated with the two basis functions forming that density. The interaction distance between two densities $\rho_{ab}$ and $\rho_{cd}$ is defined as:

(5) $$d\left(\rho_{ab},\rho_{cd}\right)=\left\|\frac{r_{a}+r_{b}}{2}-\frac{r_{c}+r_{d}}{2}\right\|,$$

where $r_{a},\ldots ,r_{d}$ are the positions of the atoms involved in the four basis functions *a*, *b*, *c*, *d*.

A lower and upper cut-off threshold $r_{c_{1}}$ and $r_{c_{u}}$ (typically 8 and 10 Å), are introduced to control the inclusion of interactions. For density pairs with distances between these two thresholds, a smooth transition is applied using a cut-off function to avoid discontinuities. The scaling function (cut-off function) used is

(6) $$f(x)=1+2x^{3}-3x^{2},$$

which smoothly interpolates between 1 and 0 as *x* increases in the interval [0,1]. The distance interval $\left[r_{c_{1}},r_{c_{\text{u}}}\right]$ is linearly mapped to the domain [0,1] of f(x).

### Electron repulsion integral algorithm

An electron repulsion integral (ERI) describes the interaction between two electronic densities:

(7) $$e_{ijkl}\equiv (ij|kl)=\int \int drdr^{\prime }\frac{\varphi_{i}(r)\varphi_{j}(r)\cdot \varphi_{k}\left(r^{\prime }\right)\varphi_{l}\left(r^{\prime }\right)}{\left\|r-r^{\prime }\right\|}\;\;=\sum_{a=1}^{n_{{\text{G}}_{i}}}\sum_{b=1}^{n_{{\text{G}}_{j}}}\sum_{c=1}^{n_{{\text{G}}_{k}}}\sum_{d=1}^{n_{{\text{G}}_{l}}}\int \int drdr^{\prime }\frac{A_{a}A_{b}A_{c}A_{d}}{\left\|r-r^{\prime }\right\|}\cdot \text{exp}\left(-\alpha_{a}{\left\|r-r_{a}\right\|}^{2}-\alpha_{b}{\left\|r-r_{b}\right\|}^{2}-\alpha_{c}{\left\|r^{\prime }-r_{c}\right\|}^{2}-\alpha_{d}{\left\|r^{\prime }-r_{d}\right\|}^{2}\right).\;\;\equiv \sum_{a=1}^{n_{{\text{G}}_{i}}}\sum_{b=1}^{n_{{\text{G}}_{j}}}\sum_{c=1}^{n_{{\text{G}}_{k}}}\sum_{d=1}^{n_{{\text{G}}_{l}}}\left(g_{a}g_{b}|g_{c}g_{d}\right),$$

where each orbital is expanded in terms of Gaussian functions. Each ERI of the individual Gaussian functions is given by:

(8) (gagb∣gcgd)=OgagbOgcgdPgagbgcgdF11(12,32,−Pgagbgcgddgagbgcgd2)

with the confluent hypergeometric function F11, two overlaps $O_{g_{a}g_{b}}$ and $O_{g_{c}g_{d}}$, a factor $P_{g_{a}g_{b}g_{c}g_{d}}$ and a distance $d_{g_{a}g_{b}g_{c}g_{d}}$ which are defined as:

(9) $$O_{g_{a}g_{b}}:=\sqrt{2}\pi^{\frac{5}{4}}A_{a}A_{b}\frac{1}{{\left(\alpha_{a}+\alpha_{b}\right)}^{\frac{3}{2}}}\cdot \text{exp}\left(-\frac{\alpha_{a}\alpha_{b}}{\alpha_{a}+\alpha_{b}}{\left\|r_{a}-r_{b}\right\|}^{2}\right),$$

(10) $$P_{g_{a}g_{b}g_{c}g_{d}}:=\frac{1}{\frac{1}{\alpha_{a}+\alpha_{b}}+\frac{1}{\alpha_{c}+\alpha_{d}}},$$

(11) $$d_{g_{a}g_{b}g_{c}g_{d}}:=\left\|r_{ab}-r_{cd}\right\|,r_{ab}=\frac{\alpha_{a}r_{a}+\alpha_{b}r_{b}}{\alpha_{a}+\alpha_{b}}.$$

We define $x:=P_{g_{a}g_{b}g_{c}g_{d}}d_{g_{a}g_{b}g_{c}g_{d}}^{2}$ and evaluate the confluent hypergeometric function using the following equation:

(12) F11(12,32,−x)=πerf(x)2x,

where the error function can be efficiently approximated using:

(13) $$\text{erf}(x)≅1-\left(c_{1}t+c_{2}t^{2}+c_{3}t^{3}+c_{4}t^{4}+c_{5}t^{5}\right)\cdot \text{exp}\left(-x^{2}\right),t=\frac{1}{1+c_{0}x}$$

(with coefficients $c_{0}=0.3275911$, $c_{1}=0.254829592$, $c_{2}=0.284496736$, $c_{3}=1.421413741$, $c_{4}=-1.453152027$ and $\left.c_{5}=1.061405429\right)$^59^. For large *x* the error function erf(x) rapidly approaches 1, since $1-erf(4)\approx 1.5\cdot 10^{-8}$ and $1-erf(5)\approx 1.5\cdot 10^{-12}$, and can be approximated accordingly.

We recall that we evaluate the ERIs of the form

(14) $$(ij|kl)=\sum_{a=1}^{n_{{\text{G}}_{i}}}\sum_{b=1}^{n_{{\text{G}}_{j}}}\sum_{c=1}^{n_{{\text{G}}_{k}}}\sum_{d=1}^{n_{{\text{G}}_{l}}}\left(g_{a}g_{b}|g_{c}g_{d}\right).$$

It will only be necessary to compute a small part of all possible combinations (ij∣kl), since we use both a density cut-off and a Coulomb cut-off. Therefore, and to reduce redundant computation, the quadruple summation over all Gaussians is reformulated as a double summation over precomputed Gaussian pairs $g_{a}g_{b}$ and $g_{c}g_{d}$, for which the quantities $O_{g_{a}g_{b}}$ and $r_{ab}$ as well as $O_{g_{c}g_{d}}$ and $r_{cd}$, respectively, are stored in lookup tables. This is practical because number of density pairs is considerably smaller than the total number of ERIs. The complete ERI evaluation algorithm can be visualized in terms of a three-dimensional tensor: the first and second axis are the sums described above and the third axis is the list of all relevant combinations (ij∣kl). Summing up this tensor along the first two axes yields the full list of ERIs. It must be noted that each integral $\left(g_{a}g_{b}|g_{c}g_{d}\right)$ in this tensor is computed by using the same equation. However, the lengths of the first two axes $n_{G_{i}}n_{G_{j}}$ and $n_{G_{k}}n_{G_{l}}$ are not always the same as the number of used Gaussian functions is not the same for all wave functions.

To account for this, we modify the equation for (ij∣kl) by setting the limits of the sums to $maxn_{G_{i}}n_{G_{j}}$ and $maxn_{G_{k}}n_{G_{l}}$ which leads to:

(15) $$(ij|kl)=\sum_{a,b=1}^{\text{max}n_{{\text{G}}_{i}}n_{{\text{G}}_{j}}}\sum_{c,d=1}^{\text{max}n_{{\text{G}}_{k}}n_{{\text{G}}_{l}}}\delta_{g_{a}g_{b}g_{c}g_{d}}^{ijkl}\left(g_{a}g_{b}|g_{c}g_{d}\right).$$

where $\delta_{g_{a}g_{b}g_{c}g_{d}}^{ijkl}$ is 1 if $ab\leq n_{G_{i}}n_{G_{j}}$ and $cd\leq n_{G_{k}}n_{G_{l}}$—otherwise it is 0 . Therefore, $\delta_{g_{a}g_{b}g_{c}g_{d}}^{ijkl}$ describes if the index combination *abcd* is defined for the set of Gaussian functions $g_{a}g_{b}g_{c}g_{d}$ within (ij∣kl).

This method allows the evaluation of all (ij∣kl) with the same equation and is therefore highly parallelizable. Additionally, it saves memory, since no intermediate values are stored (all Gaussian ERIs $\left(g_{a}g_{b}|g_{c}g_{d}\right)$ become contracted immediately in each step of the loop over (ij∣kl)). Precomputing and storing intermediate values dependent on only one density ($O_{g_{a}g_{b}}$, $O_{g_{c}g_{d}}$, $r_{ab}$ and $r_{cd}$) reduce the computational effort as well.

### Validation of cut-offs and basis

To ensure that the proposed cut-off methods and basis function manipulations do not significantly compromise the accuracy of the calculations, results were benchmarked against the quantum-chemistry package ORCA^13,60,61,62,63,64^. Specifically, we assessed the agreement between our results and a standard calculation using the STO-3G basis set, focusing on both total and orbital energies. This allowed us to quantify the impact of lobe function expansion and interaction truncations on the computed energies. As a test system, we selected the molecule beta-carotene (C_40_H_56_), which involves 541,089,856 unique ERIs, of which 30,572,290 were considered to have relevant density contributions. Among these, 3,946,243 integrals met both density and Coulomb interaction relevance criteria.

The total electronic energy calculated using our implementation was −1528.547 Hartree (Ha), compared with −1528.663 Ha obtained via ORCA. This corresponds to an absolute deviation of 0.115 Ha, or 0.0075%. For the orbital energies, we compared all valence orbitals and the ten lowest virtual orbitals, yielding a root mean square deviation of 7.98 mHa. Using only the Coulomb cut-off leads to a value of 7.57 mHa, whereas only density cut-offs result in an error of 6.07 mHa. No cut-offs at all result in a deviation of 3.23 mHa which is attributed to the modified basis functions in the lobe function expansion which do not match the STO-3G basis exactly and therefore lead to a small error.

In terms of computational performance, ORCA required 192 seconds for a beta-carotene calculation on an Intel i5 processor. In comparison, our implementation with all cut-offs was completed in 21 seconds. When disabling the Coulomb cut-off and using a minimal density threshold of 10^−10^, typical of many Hartree–Fock codes, the runtime increased to 131 seconds.

The accuracy of subsystem splitting was tested using the protein Evasin P1126 (PDB ID: AF_AFA0A023FF81F1)^42^. For atomic energy calculations, only valence electrons were considered. Subsystems were constructed by segmenting the amino acid chain of the whole protein into smaller peptide fragments. Comparing a full Hartree-Fock calculation with the subsystem-based approach, we obtained a root mean square deviation of 0.385 Ha for the atomic energies, corresponding to a relative error of 0.444%.

### Parallel Hartree–Fock on large systems

The density cut-off was set to 10^−4^ and the Coulomb cut-off to 10 Å with a smooth transition to zero starting at 8 Å. The self-consistent cycle of the Hartree–Fock calculation used a convergence threshold of 10^−6^ for the RMSD of the density matrices. The STO-3G basis set was split into individual Gaussian functions to allow an ERI evaluation according to the above-mentioned Gaussian lobe function expansion algorithm.

System preparation involved solvation in water using the solvation tool of the programme Visual Molecular Dynamics (VMD)^65^. The solvated system was then partitioned into subsystems on a 3D mesh. Hartree-Fock calculations were carried out independently for each subsystem. To reconstruct the total electronic density, the density matrix elements were chosen from the subsystem containing both atoms corresponding to the two basis functions. If the basis functions were associated with different subsystems, the mean of the relevant density matrix elements was used.

The total electronic density was then calculated as

(16) $$\rho_{\text{total}}(r)=\sum_{a=1}^{n_{\text{bf}}}\sum_{b=1}^{n_{\text{bf}}}r(a,b)P_{ab}\rho_{ab}(r),$$

where $n_{bf}$ is the total number of basis functions, r(a,b) is a relevance determining whether the pair basis functions *a* and *b* contributes significantly, and $P_{ab}$ is the corresponding element of the density matrix.

### Spectral calculation parameters

For the quantum spectral calculations, we employed the real-time time-dependent Hartree–Fock (RT-TDHF) formalism, as it scales more favourably for larger systems than the linear-response time-dependent Hartree–Fock (LR-TDHF) approach, which theoretically scales with the sixth power of the system size.

In RT-TDHF the system is excited by an electric field pulse and propagated in time using quantum-mechanical time evolution. During the propagation, the dipole moments are recorded at each time step, and the frequency-dependent polarizability and absorption spectra are obtained via discrete Fourier transform of the dipole time series. The systems were propagated over 2,000 steps with a time step of 0.25 atomic units (a.u.), corresponding to a total propagation time of 500 a.u. (12.1 fs). The applied electric field pulse had a strength of 10^−5^ a.u., a standard deviation of 0.2 a.u., and a starting time of 2 a.u. During the Fourier transform, an attenuation factor of 0.01 a.u. was used to supress numerical noise.

Following established practice, the resulting spectra were rescaled^66,67^ to account for the systematic shifts in excitation energies inherent to time-dependent Hartree–Fock and density functional theory calculations. A rescaling factor of 1.335 was applied, consistent with the benchmark study by Jacquemin et al., who computed TDHF spectra for a wide range of organic dyes^67^.

### Atomic energy processing

The atomic energies used for the comparison with AlphaFold’s pLDDT scores were smoothed using a Savitzky-Golay filter^68^ with a window length of 150. To match the scale of pLDDT values, the computed energies were multiplied by the factor of 15 and shifted by a structure-specific offset (−430, −440 and −460 for PDB entries AF_AFA0A023FF81F1^42^, PDB AF_AFA0A023IKK2F1^42^ and AF_AFA0A023GPK8F1^42^, respectively). The rescaled energies were truncated at the corresponding structure’s maximum and minimum pLDDT values to eliminate extreme atomic energy outliers.

A modified expression for atomic energies was employed, defined as

(17) $$E_{a}=\frac{1}{Z_{a}}\sum_{i=1}^{n_{{\text{bfv}}_{a}}}\sum_{j=1}^{n_{\text{bf}}}P_{ij}\left(H_{ij}+\frac{1}{2}G_{ij}\right),$$

where $E_{a}$ is the atomic energy of atom, $Z_{a}$ is the atomic charge (used for normalisation), $n_{{\text{bfv}}_{a}}$ is the number valence basis functions for atom *a* (excluding core contributions), and $n_{bf}$ is the total number of basis functions. $P_{ij}$, $H_{ij}$ and $G_{ij}$ denote the density matrix, core Hamiltonian, and two-electron interaction matrix elements, respectively.

System preparation was based on the aforementioned PDB entries^42^, with solvation performed using a 10 Å water shell generated via the solvation tool of Chimera^69^.

### Programme implementation

The described programme is written in the Python programming language^70^. To achieve high computational performance, all computational demanding functions are written either in matrix form or compiled to machine code with just-in-time (JIT) compilation. For JIT compilation, the library Numba^71^ was used, and matrix operations were performed with Numpy^72^ or PyTorch^73^. Visualisation of results and plotting were performed using the library Matplotlib^74^.

### Biomolecular structures and visualisation

All calculations in this article were based on biomolecular structures obtained from the RCSB Protein Data Bank (PDB)^42^. The structures and their full references (including original research articles and PDB entries) are as follows: PDB 2M3X^28^ (12-bladed propeller protein), PDB 1H6 8^31,75^ (rhodopsin), PDB 6Q3G^1,76^ (bacteriophage P68), PDB 1A7Y^5,77^ (Actinomycin D), PDB 1D79^3,78^ (DNA with 8 base pairs), PDB 2JYK^4,79^ (DNA with 21 base pairs), PDB 1MNV^6,80^ (Actinomycin D bound to DNA), PDB 8WL2^33,81^ (flagellar motor), PDB 7PKR^34,82^ (vault protein complex). Structures used for comparing AlphaFold’s pLDDT scores to atomic energies include: PDB AF_AFA0A023FF81F1 (Evasin P1126), PDB AF_AFA0A023IKK2F1 (MHC Class II Beta Chain) and PDB AF_AFA0A023GPK8F1 (Friend of Echinoid, Isoform H). These were obtained from the Computed Structure Models (CSM) section of the RCSB PDB^42^.

Visualisations of biomolecular structures were created using ChimeraX^29,69,83,84^. All other figure elements (including biomolecular structures at atomic resolution, electronic densities, absorption spectra and atomic energies) were generated with Matplotlib^74^ for python.

## Extended Data

**Figure 6:**
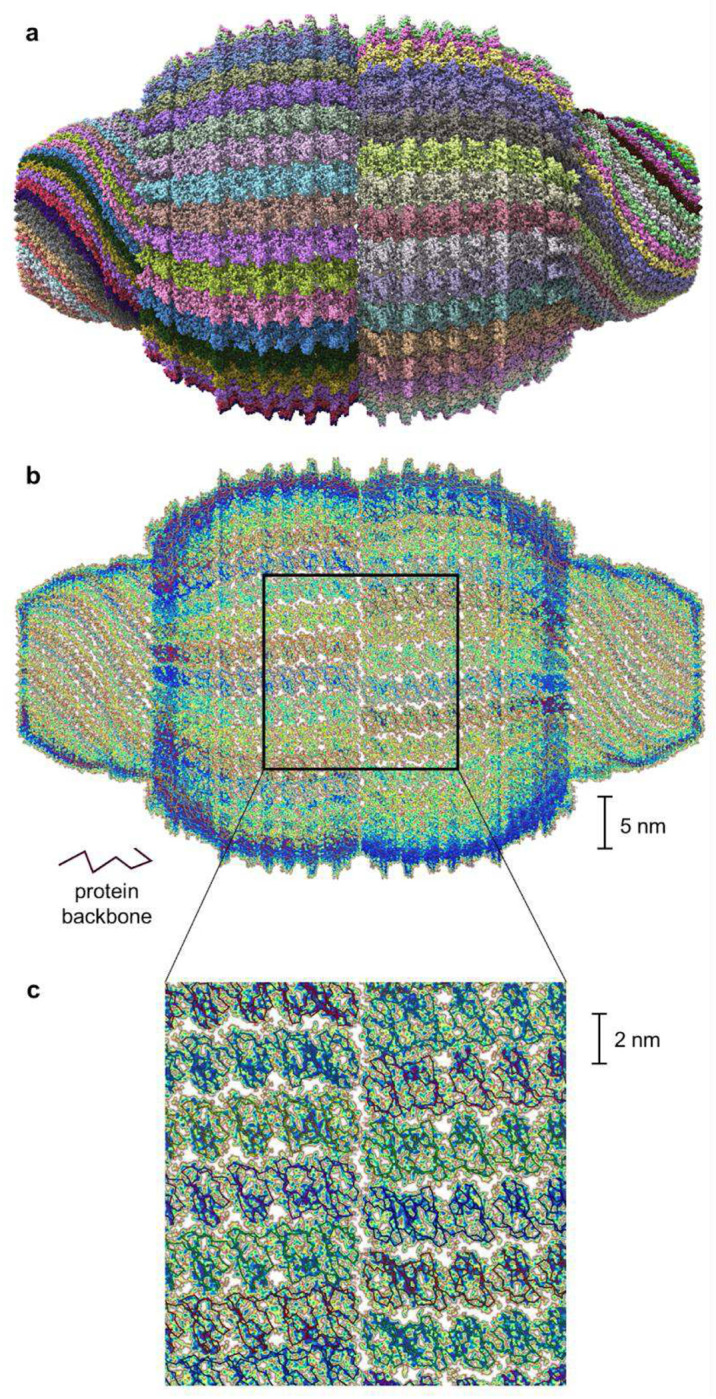
Calculated electronic density of the vault protein complex. Detailed depiction of the electronic density of the vault protein complex PDB 7PKR^34^, computed using the Hartree-Fock method presented in this work. **a**, Atomistic representation of the complete molecule. **b**, Projection of the electronic density of one half on the vault structure along the x-axis. **c**, Close-up view of the region highlighted in **b**. The protein backbone is displayed as coloured lines, with brown indicating regions of low and blue of high electronic density. For clarity, the electronic density of the solvent is omitted and only contributions from valence basis functions are shown.

**Figure 7:**
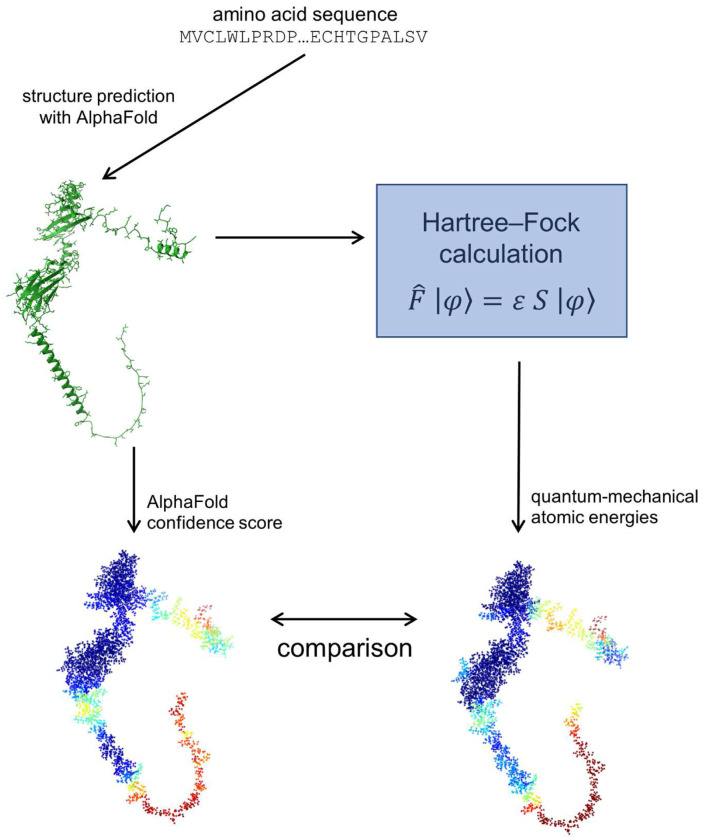
Comparison of quantum energies and pLDDT scores. Graphical abstract illustrating one of the main findings of this work: the correlation between AlphaFold’s confidence scores and quantum-mechanical atomic energies. A structure prediction from AlphaFold (based on a known amino acid sequence) is analysed using Hartree–Fock calculations to determine atomic energies. These energies are then compared with the pLDDT scores, which are a direct output from AlphaFold. The visualisation of the protein structure PDB AF_AFA0A023FF81F1 was generated using ChimeraX^29^ with data from rcsb.org^42^.

**Figure 8:**
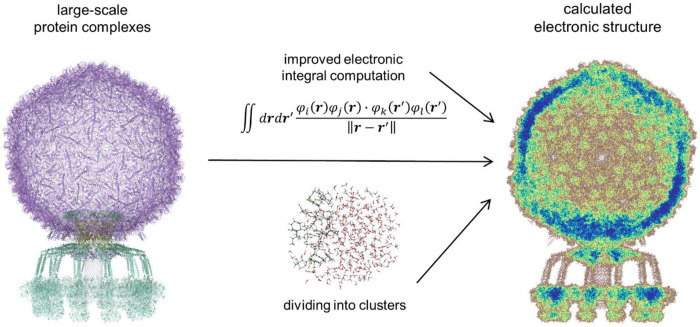
Graphical abstract for large-scale quantum-mechanical calculations. Accessing the electronic structure (right) of large-scale biological structures as the bacteriophage P68^1^ (left) is made possible by dividing the structure into clusters and employing an improved algorithm for electronic integral computation.

**Figure 9:**
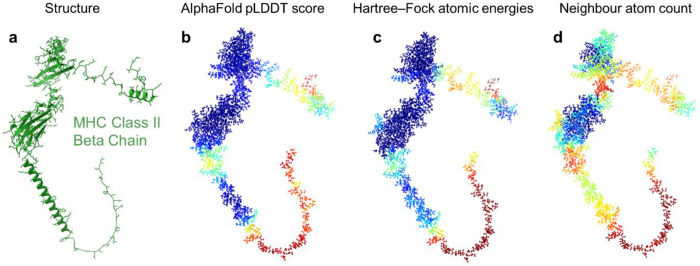
Atomic energies versus neighbouring atom count. For the predicted protein structure PDB AF_AFA0A023IKK2F1^42^ (**a**), the structure evaluation method using atomic energies from Hartree–Fock is compared with an approach that uses the number of neighbouring atoms as a metric. Higher local atom density is typically associated with more accurate predictions, resulting in a loose correlation between the neighbouring atom count (**b**) and AlphaFold’s pLDDT scores (**d**). However, the evaluation of protein structures with Hartree–Fock atomic energies (**c**) provides a more accurate assessment. For example, the alpha helix at the bottom left of the structure is correctly assigned high confidence by the quantum approach but not by the neighbour count method. Similarly, the linker between the two top-left domains is identified as stable by the Hartree–Fock energies but misclassified as a weak region by the atom density method.

**Figure 10:**
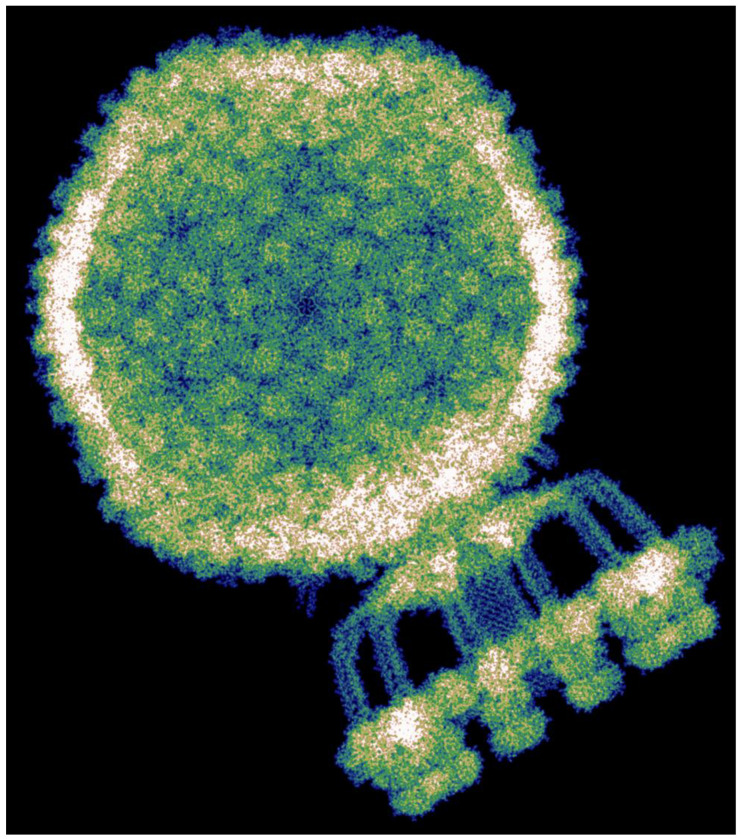
Computed electronic density of the bacteriophage P68. Detailed visualisation of the electronic density of the bacteriophage P68^1^, calculated using the presented Hartree-Fock method. Colours range from black/blue (low density) to white (high density). For improved clarity, the electronic density of the solvent is omitted, and only contributions from valence basis functions are shown.

## Data Availability

Generated data are available by the authors upon request.

## Code Availability

Code is made available by the authors upon reasonable request.
